# Proteomic analyses reveal misregulation of LIN28 expression and delayed timing of glial differentiation in human iPS cells with *MECP2* loss-of-function

**DOI:** 10.1371/journal.pone.0212553

**Published:** 2019-02-21

**Authors:** Jean J. Kim, Jeffrey N. Savas, Meghan T. Miller, Xindao Hu, Cassiano Carromeu, Mathieu Lavallée-Adam, Beatriz C. G. Freitas, Alysson R. Muotri, John R. Yates, Anirvan Ghosh

**Affiliations:** 1 Section of Neurobiology, Division of Biological Sciences, University of California San Diego, La Jolla, California, United States of America; 2 Department of Pediatrics, University of California San Diego, La Jolla, California, United States of America; 3 Department of Chemical Physiology, The Scripps Research Institute, La Jolla, California, United States of America; 4 Pharma Research and Early Development (pRED), Roche Innovation Center Basel, F. Hoffmann-La Roche, Basel, Switzerland; University of Minnesota Medical Center, UNITED STATES

## Abstract

Rett syndrome (RTT) is a pervasive developmental disorder caused by mutations in *MECP2*. Complete loss of *MECP2* function in males causes congenital encephalopathy, neurodevelopmental arrest, and early lethality. Induced pluripotent stem cell (iPSC) lines from male patients harboring mutations in *MECP2*, along with control lines from their unaffected fathers, give us an opportunity to identify some of the earliest cellular and molecular changes associated with *MECP2* loss-of-function (LOF). We differentiated iPSC-derived neural progenitor cells (NPCs) using retinoic acid (RA) and found that astrocyte differentiation is perturbed in iPSC lines derived from two different patients. Using highly stringent quantitative proteomic analyses, we found that *LIN28*, a gene important for cell fate regulation and developmental timing, is upregulated in mutant NPCs compared to WT controls. Overexpression of LIN28 protein in control NPCs suppressed astrocyte differentiation and reduced neuronal synapse density, whereas downregulation of LIN28 expression in mutant NPCs partially rescued this synaptic deficiency. These results indicate that the pathophysiology of RTT may be caused in part by misregulation of developmental timing in neural progenitors, and the subsequent consequences of this disruption on neuronal and glial differentiation.

## Introduction

In RTT, female patients are heterozygous for mutations in *MECP2* and therefore display mosaicism due to either balanced or non-balanced X-inactivation[1, 2]. In rare cases, male patients with *MECP2* mutations have survived to term and beyond[3]. In such patients, the MECP2 protein is either entirely absent or is significantly mutated in all cells. Male MECP2 deficiency manifests as neonatal encephalopathy, accompanied by deficits in dendritic arborization and synaptic spines in the neocortex, and death during the first few years of life[4].

Human iPSCs are emerging as a powerful experimental paradigm to model complex human neurological disorders[5–10]. Models of RTT using female patient-derived iPSCs and genome-edited human embryonic stem cells (hESCs) have been useful in highlighting deficits in neuronal synapse maturation and activity[5, 6, 9–11], both hallmarks of cells harboring *MECP2* mutations *in vivo* [12–14]. Additionally, in mouse models, glia have been shown to actively contribute to the pathophysiology of RTT [15–18]. However, how *MECP2* mutations affect glial development is not well understood.

We used male patient-derived iPSCs as an *in vitro* human disease model of complete *MECP2* LOF to identify molecular pathways that may underlie the cellular pathophysiology of RTT. We derived forebrain progenitors and forebrain neurons by directed differentiation of human pluripotent stem cells (hPSCs) from two patient samples[19, 20]. Following RA treatment, we observed profound differences in the ability of *MECP2* mutant NPCs to differentiate into GFAP-positive glia. By combining isotopic protein labeling with mass spectrometry, we found significantly reduced levels of many astrocytic markers in mutant cultures. In additional proteomic experiments, we identified abnormal upregulation of LIN28 in mutant NPCs. As LIN28 is a known developmental driver important for neural differentiation and its expression level was inversely correlated with the ability to generate GFAP-positive glia[21, 22], we hypothesized that LIN28 is regulated by MECP2 and that its misregulation in patient NPCs may affect astrocytic differentiation.

## Results

### *MECP2*-mutant NPCs show perturbed neuronal and glial differentiation

**Fig 1:**
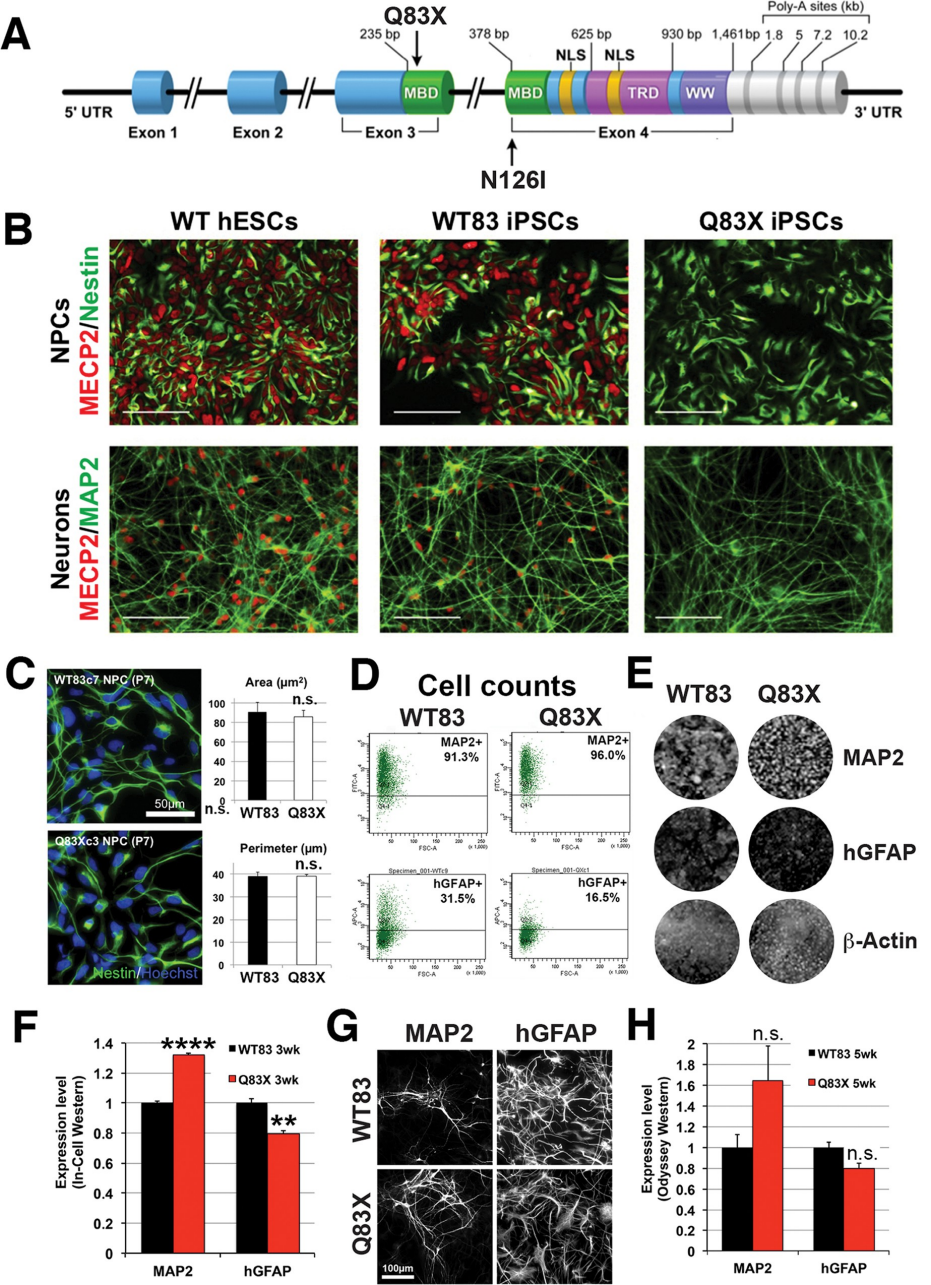
NPCs and neuronal cultures derived from male patients with *MECP2* mutations and their unaffected fathers show differential neuron-to-glia ratios. A. Diagram of MECP2 showing the relative positions of mutations found in Q83X and N126I patients.B. MECP2 staining (in red) in NPCs and neurons derived from WT hESCs, WT83 iPSCs, and Q83X iPSCs. Upper panels, NPCs were stained with anti-Nestin antibodies (in green), and lower panels, neurons with anti-MAP2 antibodies (in green).C. Comparison of average nuclear area and perimeter in WT83 and Q83X NPCs. NPCs were stained with anti-Nestin antibodies (in green), and nuclei with Hoechst 33342 (in blue). Scale bar is 50 μm. *p* = 0.342 by a one-tailed unpaired *t*-test. All *p*-values >0.05 are labeled as “n.s.” (not significant).D. Flow cytometry data showing percentage of MAP2+ and GFAP+ cells in 3-week-old RA-treated cultures from WT83 and Q83X NPCs.E. Representative In-Cell Western images showing MAP2, GFAP, and beta-Actin staining in 3-week-old RA-treated WT83 and Q83X NPCs. F. Quantification of the In-Cell Western experiment shown in E. ** *p*-value <0.02 and **** *p*-value <0.0001 by a two-tailed unpaired *t*-test.G. Immunofluorescence images showing MAP2 and GFAP expression in 5-week-old RA-treated cultures from WT83 and Q83X NPCs. Scale bar is 100 μm. H. Quantification of Odyssey Western blots showing relative MAP2 and GFAP expression in protein extracts from 5-week-old RA-treated cultures from WT83 and Q83X NPCs. *p* = 0.108 for MAP2 and *p* = 0.051 for GFAP.

To investigate how MECP2 deficiency affects neural differentiation *in vitro*, we used previously generated and characterized iPSC lines reprogrammed from two male patients, with either an early termination of *MECP2* translation (Q83X) or a polar-to-hydrophobic amino acid substitution in the methyl-CpG-binding domain (N126I) (Fig 1A) [11]. We used iPSCs derived from their respective unaffected fathers (WT83 and WT126) as controls, and all NPCs were differentiated by directed differentiation in serum-free conditions[19]. The absence of MECP2 expression in Q83X mutant NPCs and neurons was verified by immunofluorescence (Fig 1B). Smaller soma[5, 23, 24] and smaller nuclei[9, 25] have been observed in MECP2-deficient neurons compared to WT. However, NPCs derived from *MECP2*-mutant patients iPSCs appeared to be similar to controls and did not show reduced nuclear size (Fig 1C), consistent with a previous report that nuclei were smaller in mutant neurons but not in NPCs derived from genome-edited *MECP2*-mutant hESCs[9].

Following neural differentiation of control and *MECP2*-mutant cultures, we observed persistent differences in the expression levels of two commonly used markers for neurons and glia, MAP2 and GFAP (Fig 1D–1H). Analysis of cell populations by flow cytometry revealed a slightly higher percentage of MAP2-expressing cells and a lower percentage of GFAP-expressing cells in Q83X cultures compared to WT83 cultures (Fig 1D; S1A Fig). A clear reduction in GFAP-positive cells was also observed in the N126I-mutant culture, although the percentage of MAP2-positive cells was also somewhat reduced (S1B Fig). We used ECL-based Western blots, immunofluorescence staining, infrared fluorescence (IRFL)-based Odyssey Western blots, and IRFL-based In-Cell Westerns to quantitate the differences in Q83X cultures (Fig 1E–1H). We consistently observed increased MAP2 and decreased GFAP expression levels in Q83X-mutant lines at 3 weeks after RA treatment (Fig 1F), indicating increased neuron-to-glia ratios in mutant cultures compared to WT. After 5 weeks of culture, the differences in lysates were no longer statistically significant, but there was a clear trend of increased neuronal MAP2 and decreased glial GFAP expression that supported our other results from 3-week cultures (Fig 1H).

### SILAC and quantitative proteomic screening reveals astrocyte markers are decreased in *MECP2*-mutant NPCs

**Fig 2:**
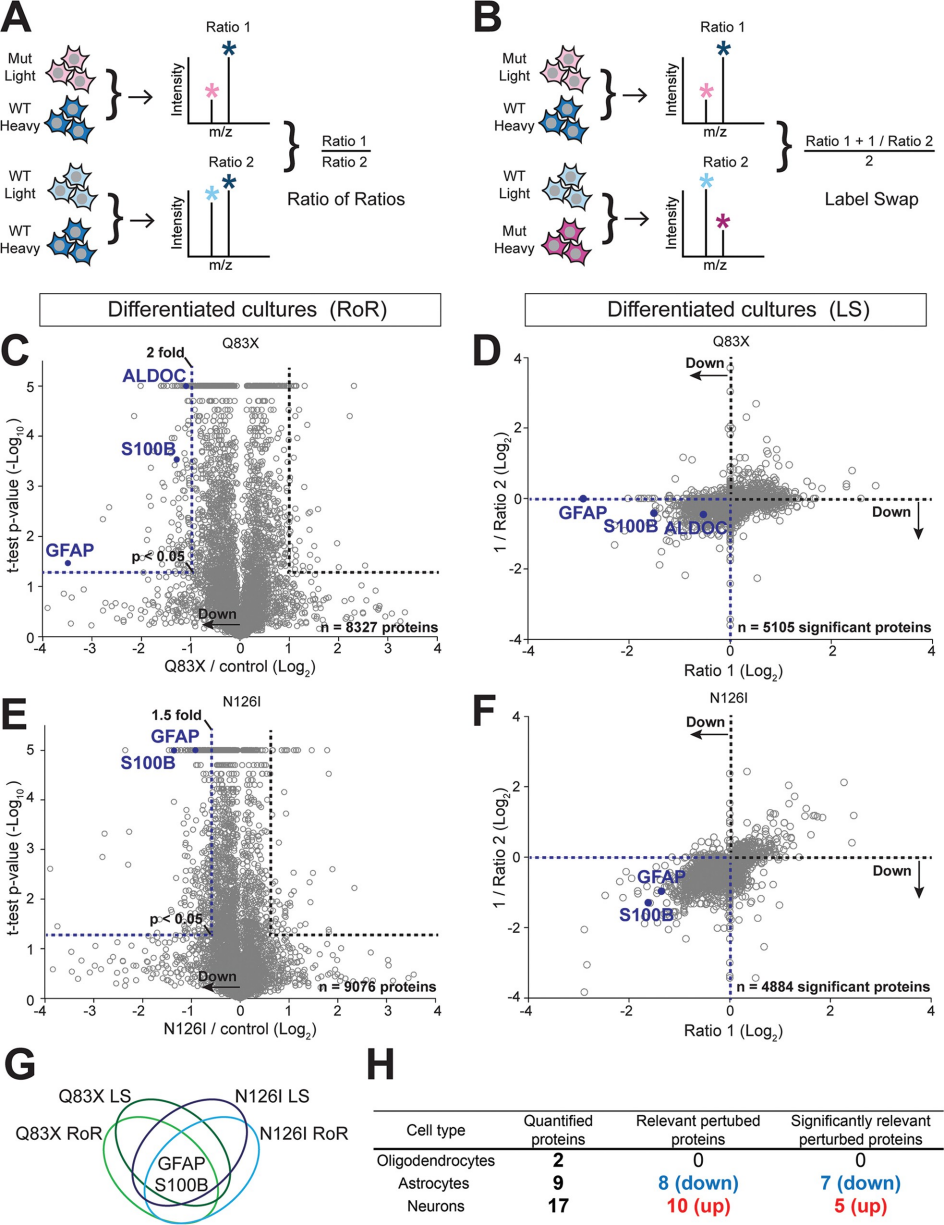
Deep quantitative proteomic analyses of SILAC-labeled WT and *MECP2* mutant (Mut) 3-week-old differentiated cultures. A. Diagram of the Ratio of Ratios (RoR) paradigm. “Heavy” denotes SILAC labeled (Arg+10 and Lys+8) cultures. “Light” denotes unlabeled cultures. N ≥ 3 cultures. B. Diagram of the Label Swap (LS) paradigm. N ≥ 4 cultures. C. Volcano plot showing confidently quantified proteins by LC-MS in WT83 and Q83X comparison by the RoR paradigm. The blue dotted line demarcates proteins that have a *t*-test *p*-value < 0.05 (FDR = 0.14) and that are downregulated by at least 2-fold. N = 8327 proteins.D. Scatter plot showing all confidently quantified proteins by LC-MS in WT83 and Q83X comparison by the LS paradigm that are significantly differentially expressed. N = 5105 significant proteins, *t*-test *p*-value < 0.05 (FDR = 0.14). E. Volcano plot showing confidently quantified proteins by LC-MS in WT126 and N126I comparison by the RoR paradigm. The blue dotted line demarcates proteins that have a *t*-test *p*-value < 0.05 (FDR = 0.17) and that are downregulated by at least 1.5-fold. N = 9076 proteins. F. Scatter plot showing all confidently quantified proteins by LC-MS in WT126 and N126I comparison by the LS paradigm that are significantly differentially expressed. N = 4884 significant proteins, *t*-test *p*-value < 0.05 (FDR = 0.14).G. Schematic diagram showing the identification of GFAP and S100B at the intersection of the two SILAC approaches (RoR and LS) and the two independent *MECP2* mutations (Q83X and N126I).H. Summary of the numbers of neural cell type-specific proteins quantified in the RoR and LS datasets.

To identify the molecular changes underlying this decreased ability for *MECP2*-mutant NPCs to differentiate into glia, we applied stable isotope labeling by amino acids in cell culture (SILAC) with quantitative multidimensional protein identification technology (MudPIT) mass spectrometry (MS)-based shotgun proteomics[26–28]. By metabolically incorporating heavy isotopically labeled amino acids into the synthesized proteins, one culture produces a “heavy” version of each protein, allowing the mass spectrometer to discriminate between “heavy” labeled proteins from one culture and unlabeled “light” proteins from a second culture (Fig 2A and 2B). By mixing the “light” and “heavy” extracts in a 1:1 ratio, we quantitatively compared their proteomes. SILAC labeling of iPSC cultures that had been terminally differentiated with RA treatment had no overt effect on neuronal morphology (S2A Fig) and all cultures displayed > 94% peptide labeling efficiency (S2B Fig).

MECP2 has been reported to regulate gene expression at multiple levels including transcriptional, post-transcriptional, and RNA processing levels[9, 29, 30]. Therefore, we hypothesized that *MECP2* mutations cause quantifiable changes in the proteomes of iPSC-derived neurons compared to WT neurons. Indeed, our proteomic analysis unveiled hundreds of perturbed proteins (Fig 2). In order to narrow our focus to only the proteins most perturbed in both *MECP2* mutant populations, we used two distinct analytical paradigms, both of which involved determining ratios of “light” to “heavy” proteins to calculate the Mutant / WT ratio. In the Ratio of Ratios (RoR) paradigm (Fig 2A), quantified proteins are normalized using a common internal standard that can accurately correct for incomplete labeling and other instrument-based biases[31]. In the Label Swap (LS) paradigm (Fig 2B), we generated two ratios for each protein from four samples[32, 33]. In this way, we focused on those proteins that were significantly altered in both RTT affected son / paternal control comparisons.

We first analyzed proteomes of the Q83X patient relative to the paternal WT83 control with the RoR paradigm and found three proteins—ALDOC, S100B, and GFAP—were all downregulated by at least 2-fold (*p*-value <0.05; Fig 2C). Next, we investigated if these proteins were downregulated in both ratios with a *p* value <0.05 using the LS approach. All three key astrocytic markers were expressed at lower levels in the Q83X cultures (Fig 2D). We then tested N126I iPSCs and consistently found that two (GFAP and S100B) of the three key astrocytic markers had at least a 1.5-fold decrease in expression and a *t*-test *p*-value <0.05 (Fig 2E). Lastly, we examined the proteomes of N126I mutant cells with the LS paradigm and again found that GFAP and S100B met our inclusion criteria (Fig 2F). The joint probability that a protein is found downregulated by chance using all of our inclusion criteria in both Q83X and N126I cells is estimated to be 0.0018 (Fig 2G; refer to **Statistical analysis of SILAC results** in the Materials and methods section).

To investigate whether or not astrocytes were selectively affected, we systematically searched for proteins that were mapped to known neural cell type-specific genes in our proteomic datasets (Fig 2H; S2C Fig, S1 Table). The cell type-specific genes were based on published data generated from cell type-specific FACS of postnatal mouse forebrain and transcriptome analysis[34]. Overall, both neuronal and astrocytic proteins were affected in both the Q83X and N126I mutant cultures differentiated with RA. Furthermore, we also made intersectional lists of perturbed proteins found in our datasets with markers that had been identified in human cortical spheroid cultures (hCS) derived from iPSCs[35] (S5 Fig, S1 Table). In that study by Sloan *et al*., hCS-derived astrocytes were immunopanned using HepaCAM, transcriptome-profiled between Day 96 and Day 495, and were clustered into Early, Middle, and Late pseudotimes. The proteins we identified remarkably spanned all pseudotimes (S6A–S6C Fig), as well as some mature astrocyte markers that overlapped with human primary astrocytes (S5D Fig). Interestingly, even though our cultures had only been differentiated for 21 days, we found perturbations of numerous astrocyte proteins in the mutant cultures that were enriched relatively late in hCS cultures and in mature astrocytes. Altogether, these discovery-based quantitative proteomic analyses demonstrate that multiple astrocytic markers are significantly downregulated in neural cultures from two distinct male RTT patient-derived iPSC lines.

### *MECP2*-mutant NPCs aberrantly regulate LIN28

**Fig 3:**
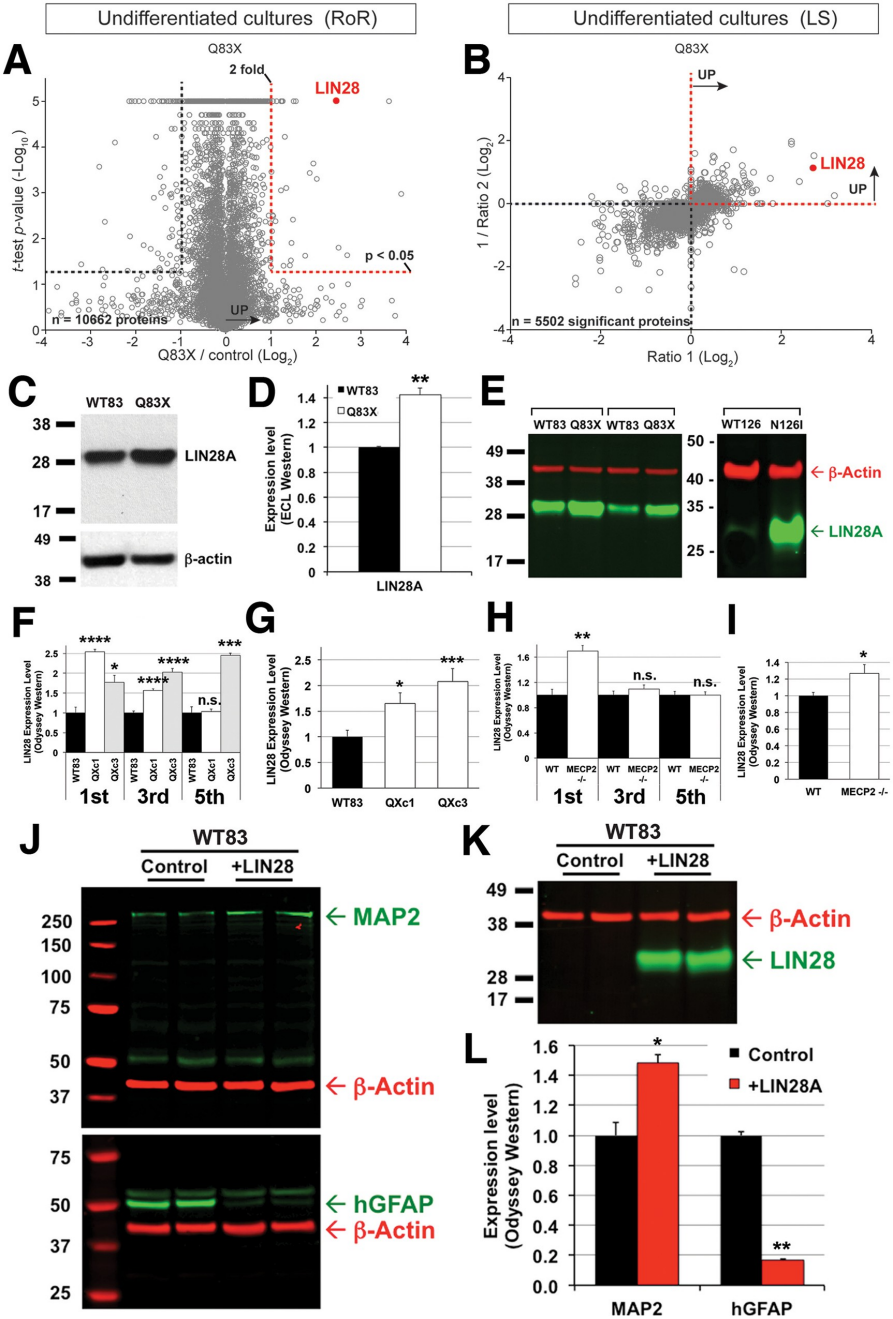
LIN28 expression is elevated in *MECP2* mutant NPCs and results in suppression of GFAP expression. A. Volcano plot showing confidently quantified proteins by LC-MS in WT83 and Q83X comparison by the RoR paradigm. The red dotted line demarcates proteins that have a *t*-test *p*-value < 0.05 (FDR = 0.15) and that are upregulated by at least 2-fold. N = 10662 proteins. B. Scatter plot showing all confidently quantified proteins by LC-MS in WT83 and Q83X comparison by the LS paradigm that are significantly differentially expressed. N = 5502 significant proteins, *t*-test *p*-value < 0.05 (FDR = 0.15). C. Enhanced chemiluminescence (ECL)-based Western blot of LIN28 expression in WT83 and Q83X NPC extracts. D. Quantification of ECL-based Western blot bands. For all bar graphs in D-L, * *p*-value <0.05, ** *p*-value <0.02, *** *p*-value <0.001, and **** *p*-value <0.0001 by a two-tailed unpaired *t*-test. E. IRFL-based Odyssey Western blot of LIN28 (green) and beta-Actin (red) expression in WT83, Q83X, WT126, and N126I NPC extracts. For WT83 and Q83X, two different sets of clones were used. F. Quantification of LIN28 expression using Odyssey Western blots in WT83 and two clones of Q83X NPCs harvested on the 1st, 3rd, and 5th passages after thawing. G. Averaged LIN28 expression in the three NPC passages shown in F. H. Quantification of LIN28 expression using Odyssey Western blots in WT and *MECP2* -/- hESC-derived NPCs harvested on the 1st, 3rd, and 5th passages after thawing. I. Averaged LIN28 expression in the three NPC passages shown in H. J.Odyssey Western blots showing expression of MAP2 (upper blot, green), GFAP (lower blot, green), and beta-Actin (red), in uninfected (Control) or LIN28-overexpressing (+LIN28) WT83 NPCs after 3 weeks of differentiation. K. Odyssey Western blots showing expression of LIN28 (green) and beta-Actin (red), in uninfected (Control) or LIN28-overexpressing (+LIN28) WT83 NPCs after 3 weeks of differentiation. L. Quantification of IRFL-based Western blot bands for MAP2 and GFAP shown in J.

Protein level changes at the NPC stage can affect major signaling pathways crucial for terminal cell fate decisions. We hypothesized that these changes could account for the differences in astrocyte markers observed in our *MECP2*-mutant differentiated cultures. Using our intersectional SILAC approach to compare the proteomes of undifferentiated WT83 and Q83X NPCs, we found that LIN28, a gene previously implicated in repressing glial differentiation, was one of the most highly and consistently upregulated proteins by both the RoR and LS approaches (Fig 3A and 3B). To rule out variability that can be introduced during directed differentiation, we verified that all NPC lines used were >90% NCAM-positive, and only ~10% p75-positive (S3A Fig). SILAC labeling of Q83X and N126I cultures had no overt effect on NPC morphology (S3B Fig) and all cultures displayed >95% peptide labeling efficiency (S3C Fig).

We verified significantly increased LIN28 expression in multiple clonal lines of Q83X using traditional and Odyssey Western blots (Fig 3C–3G). Remarkably, N126I NPCs also displayed high LIN28 expression compared to passage-matched WT NPCs (Fig 3E, right blot). We also compared the expression of LIN28 at different NPC passages and found that by passage 8, LIN28 expression could not be detected by ECL (S3D Fig). Using a slightly modified culture protocol, NPCs can be propagated stably over >20 passages with the addition of BDNF (see **Establishment of stable NPCs and serial passaging** in the Materials and methods section). We used the Odyssey Western to compare LIN28 expression levels in serially passaged NPCs, and observed that LIN28 was clearly upregulated in the Q83X clones (Fig 3F and 3G; S3E Fig). To address the concern that the age of the control did not match the probands and that age-related epigenetic factors may play a role in the expression of LIN28, we also generated isogenic genome-edited *MECP2* -/- hESCs, which were similarly differentiated and serially passaged as NPCs (S6 Fig). At the first passage assayed, *MECP2* -/- NPCs had significantly higher levels of LIN28 compared to passage-matched isogenic WT NPCs (Fig 3H). However, expression decreased to WT levels in later passages (Fig 3I). These analyses clearly show that the expression of LIN28 is significantly upregulated in NPCs that lack MECP2 function. However, it is important to note that the serial passaging experiments show that LIN28 expression changes dynamically over time in culture. Indeed, another study using hPSC-derived NPCs demonstrated a similar phenomenon[36]. Thus, *MECP2*-mutant NPCs may be unable to regulate LIN28 expression effectively over time relative to WT NPCs at equivalent passages.

### Overexpression of LIN28 represses glial differentiation in *MECP2*-mutant NPCs

LIN28 overexpression can repress glial differentiation in mouse embryonal carcinoma cells treated with RA[21]. Persistent expression of LIN28 in hPSC-derived neural progenitors has also been linked to inefficient glial differentiation in a different study[22], suggesting that elevated levels of LIN28 expression in *MECP2*-mutant NPCs could account for the observed astrocyte phenotype. To test this hypothesis, we overexpressed LIN28 in NPCs using a LIN28-expressing lentivirus (Fig 3K), and then further differentiated them for 3 weeks. In these cells, we found that MAP2 and GFAP expression were skewed in the same direction as in Q83X cultures (Fig 3J and 3L). Viral LIN28 expression persisted in the 3-week cultures without any adverse effects on viability or morphology (Fig 3K). In contrast, in our control iPSC lines, RA treatment following withdrawal of mitogens induced robust glial differentiation in NPCs. Furthermore, we found that GFAP expression was suppressed and MAP2 expression was enhanced more dramatically after forced LIN28 expression in WT83 cultures compared to mutant Q83X cultures (Fig 3L and Fig 1F, respectively).

### LIN28 overexpression decreases synapse formation in *MECP2*-mutant neurons

**Fig 4:**
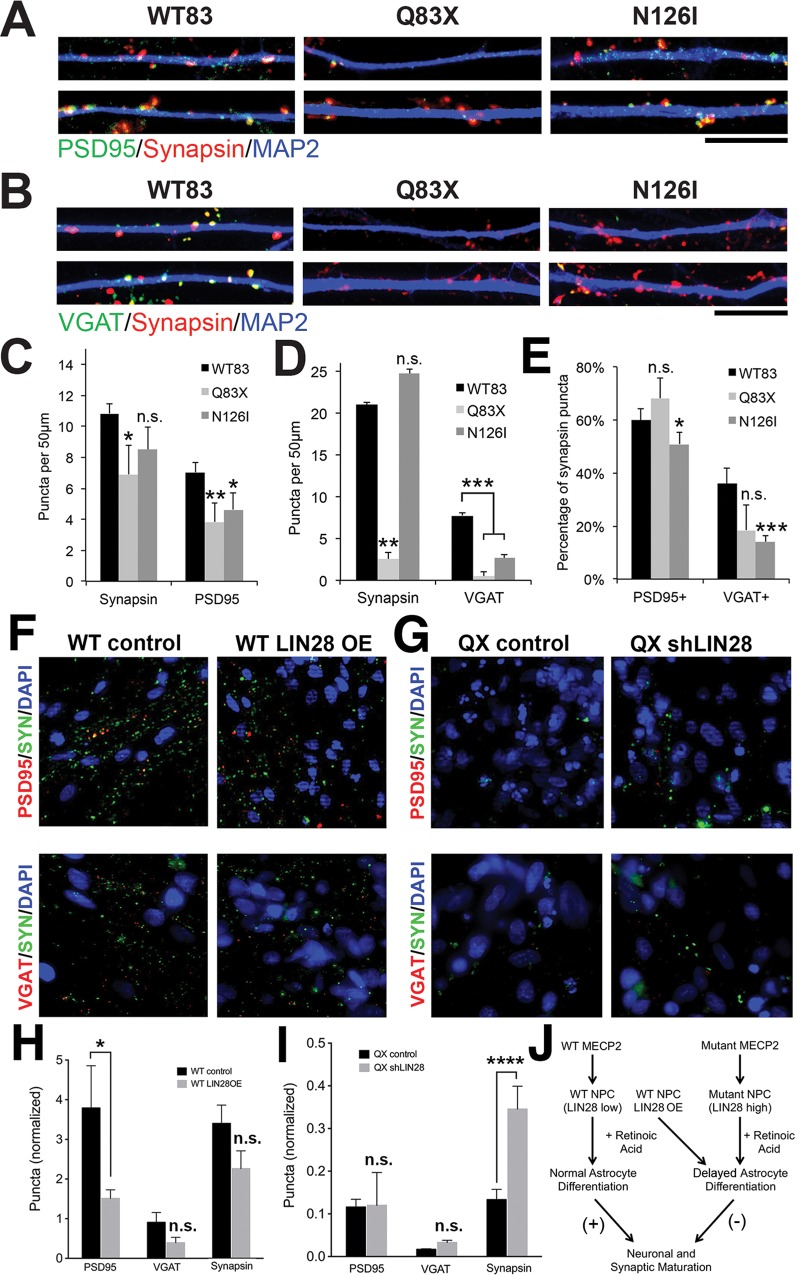
Early manipulation of LIN28 expression in NPCs alters synaptic density after terminal differentiation. A. Excitatory synaptic staining (PSD95, green) in WT83, Q83X, and N126I neurons after 5 weeks of differentiation. Two representative dendritic segments are shown per genotype. Scale bar is 10 μm. B. Inhibitory synaptic staining in cultures as in (A), except green is VGAT staining. C. Quantification of synaptic density in cultures shown in (A). Synapsin-positive puncta that were excitatory (PSD95+) were quantified in WT83 (black), Q83X (light gray), and N126I (dark gray) neurons. For all bar graphs in C-I, * *p*-value <0.05, ** *p*-value <0.02, *** *p*-value <0.001, and **** *p*-value <0.0001 by a one-tailed unpaired *t*-test. D. Quantification of synaptic density in cultures shown in (B). Synapsin-positive puncta that were inhibitory (VGAT+) were quantified in WT83 (black), Q83X (light gray), and N126I (dark gray) neurons. E. Percentage of Synapsin-positive puncta that were either excitatory (PSD95+) or inhibitory (VGAT+) in WT83 (black), Q83X (light gray), and N126I (dark gray) neurons. F. Excitatory and inhibitory synapse staining in WT83 NPCs that were differentiated for 5 weeks. WT83 NPCs were either uninfected (WT control, black) or infected with a LIN28-overexpressing lentivirus (WT LIN28 OE, gray). G. Excitatory and inhibitory synapse staining in Q83X NPCs that were differentiated for 5 weeks. Q83X NPCs were either infected with a control pLKO.1 lentivirus (QX control, black) or shRNA for LIN28 (QX shLIN28, gray). H. Normalized synaptic densities shown in (F). I. Normalized synaptic densities shown in (G). J. Schematic of the consequences of MECP2 LOF and LIN28 misregulation in mutant NPCs, leading to delayed timing of astrocyte differentiation and negative effects on neuronal synaptic maturation. LIN28 OE may phenocopy by suppressing astrocyte differentiation.

Neurons differentiated from RTT patient NPCs have been reported to display deficits in synapse development[5]. We also observed that SILAC analysis in the 3-week-old neuronal cultures also identified downregulation of neuron-specific proteins such as SNAP25, STMN2, CALB1, and SYT1 (S2C Fig). As astrocytic signals regulate synapse formation[37–39], we next wanted to determine whether neurons derived from our mutant lines had a defect in synapse formation. Therefore, we quantified excitatory and inhibitory synapse densities after 5 weeks of culture (Fig 4A–4D). Q83X neurons displayed significantly reduced Synapsin-positive presynaptic puncta, whereas N126I neurons did not show a consistent trend based on Synapsin quantification alone (Fig 4C and 4D). We also compared excitatory and inhibitory synapse numbers in WT83, Q83X, and N126I cultures by quantifying numbers of PSD95-positive puncta and VGAT-positive puncta co-localized with Synapsin. We found that both excitatory and inhibitory synapses were significantly reduced in Q83X and N126I cultures compared to WT83 (Fig 4C and 4D). By comparing the percentage of Synapsin puncta that either co-localized with PSD95 or VGAT, we observed that VGAT-positive inhibitory puncta were more significantly reduced compared to PSD95-stained glutamatergic puncta in the mutant cultures (Fig 4E), indicating that our patient iPSC-derived neurons are capable of organizing into synaptic puncta when presynaptic vesicles are present, but exhibit an overall deficiency in synaptic maturation at both excitatory and inhibitory synapses.

Since LIN28 overexpression in WT NPCs could alter proportions of neuronal versus glial differentiation, we wanted to follow the consequence of LIN28 misregulation in more mature differentiated cultures. We found that WT83 cultures overexpressing LIN28 had reduced synaptic densities compared to WT83 neurons (Fig 4F and 4H). This synaptic reduction was most significant in PSD95-positive excitatory synapses (Fig 4H), though there was also a trend of reduction in VGAT-positive inhibitory synapses and Synapsin puncta. Thus, LIN28 overexpression can phenocopy the reduced synaptic density observed in *MECP2*-mutant neurons.

Conversely, we knocked down LIN28 expression in order to test whether it was sufficient to rescue neuronal synapses. When we introduced LIN28 shRNA in Q83X NPCs, the overall density of Synapsin puncta increased significantly (Fig 4G and 4I). However, these “new” puncta appeared less mature and were not significantly labeled with either PSD95 or VGAT (Fig 4I). Thus, LIN28 misregulation at the progenitor stage may contribute to the astrocytic and synaptic phenotype observed in cultures derived from *MECP2* mutant human iPSCs.

### MECP2 interacts with the LIN28 promoter

A previous ChIP-seq study had identified an interaction of the REST complex with the *LIN28* promoter[40]. Given that MECP2 is recruited by REST as a co-repressor[41], we reasoned that MECP2 could also associate with the *LIN28* promoter. We performed chromatin immunoprecipitation of MECP2-bound genomic DNA in both WT83 iPSCs and NPCs, and found that indeed the MECP2 ChIP interaction was enriched >20-fold compared to control IgG in both cell types (S4 Fig). Enrichment of MECP2 to promoter regions in iPSCs is not that surprising as MECP2 protein expression is detectable in undifferentiated hPSCs[5, 9, 42], and in neurons, where MECP2 is highly expressed, it is thought to bind to DNA nearly as abundantly as the histone octamer[43]. The directionality of the target gene expression is likely to be context-dependent. The level of enrichment observed is comparable to MECP2 recruitment to the *BDNF* promoter, a known target of MECP2[44]. These results further strengthen our hypothesis that MECP2 directly regulates LIN28 expression in NPCs, which may in turn contribute to the glial defect during terminal differentiation of *MECP2*-mutant NPCs. At present, whether the interaction of MECP2 with the *LIN28* promoter is direct or indirect via REST remains unclear. Future molecular studies are required to explore additional MECP2-binding sites on the LIN28 promoter and interrogate its epigenetic status before and after RA treatment.

## Discussion

In this study, we found that male RTT patient-derived NPCs treated with RA skew toward neuron differentiation at the expense of glial cells. Using an unbiased discovery-based proteomic approach, we found that overexpression of LIN28 during this early proliferation may be responsible for the observed astrocyte-deficit. LIN28 has previously been linked to progenitor self-renewal and neuron-to-glia cell fate decisions[21, 22, 36, 45, 46]. Here, we saw that overexpression of LIN28 in wild type (WT) NPCs suppressed glial differentiation and led to decreased synaptic densities in WT neuronal cultures. Conversely, knockdown of LIN28 expression in Q83X NPCs was able to partially reverse synaptic deficits in the mutant lines. Altogether, our data suggest that *MECP2*-mutant cells improperly regulate LIN28 and indicate that disrupting the timing of glial differentiation may contribute to the neurological phenotype of complete *MECP2* loss-of-function.

Our results from RTT patient-derived iPSCs suggest that a defect in timing of cell fate regulation and/or response to external cues may be involved in the early pathophysiology of RTT. *MECP2* LOF in NPCs may influence very early cell differentiation timing genes such as LIN28. Interestingly, there is evidence showing that the outcome of RA treatment depends on the developmental stage of the neural progenitors (perhaps determined by the levels of timing-related genes)—RA acts to suppress astrocyte differentiation in early E13 rat progenitors and induces astrocyte differentiation in later E17 progenitors[47]. We now implicate MECP2 in the regulation of LIN28 expression in NPCs, where the LIN28 expression level is elevated and declines rapidly as differentiation occurs, allowing glial differentiation to proceed. In more advanced stages of development or in adulthood, glial gene regulation may be controlled by other factors, including MECP2 itself, which has been shown to directly regulate gene expression in postnatally cultured astrocytes[48].

Post-mortem patient brain studies may have overlooked the contribution of glial cells in the pathophysiology of RTT, because differences in glial cell number or morphology are difficult to assess accurately in patient brains. Other published protocols used to differentiate hPSCs do not produce significant amounts of glia (~10%) and often rely on co-cultures with glial feeders. We found that our differentiation method reliably generates a significant proportion (~30%) of GFAP+ astrocytes in WT iPSCs cultures following RA treatment, allowing us to observe detectable reductions in GFAP expression in our cultures.

Increased levels of MECP2 expression in patients with genomic duplications cause a different autism spectrum disorder, MECP2 Duplication Syndrome, which has shared features of RTT[49–52]. Major phenotypic abnormalities can be rescued genetically in mouse models of both RTT[53] and MECP2 Duplication Syndrome[54]. As the mechanism regulating LIN28 expression may be highly sensitive to MECP2 levels, whether LIN28 regulation is affected in the MECP2 Duplication Syndrome patient-derived NPCs remains an open avenue for investigation.

Failure of proper LIN28 regulation could result in defects in neuronal maturation and delays in glial differentiation, impairing neurodevelopmental trajectories (Fig 4J). Our data suggests that the early stage of balanced neuron/glia differentiation is important to ensure the proper course of synapse formation and maturation of neurons in the brain. Astrocytes have been shown as indispensable regulators of neuronal development[38]. We propose a mechanism whereby in the absence of MECP2 function during early neural development, LIN28 is misregulated in neural progenitors, resulting in delayed cell responsiveness to external cues and neuron/glia differentiation. This defect in astrocyte differentiation in turn contributes to the functional immaturity of neurons in the *MECP2*-mutant brain. MECP2 may regulate gene expression in various ways depending on the developmental stage. For example, one study showed that cell type- or developmental age-dependent methylation at gene promoters may be a factor in the responsiveness to extracellular signals[55]. We know from previous studies using these RTT patient iPSCs that neuronal gene expression is also altered and that the developmental timing-dependent GABA functional switch is delayed through deficits in KCC2 expression[11]. Therefore, several types of developmental timing events may be impaired in the *MECP2*-mutant cells due to improper gene regulation, leading to pervasive dysfunction in the nervous system.

## Conclusion

Our unbiased discovery-based proteomic approach identified a molecular change in male RTT patient NPCs that may contribute to the astrocytic and neuronal deficits that are observed in subsequent terminally differentiated cultures, and demonstrate the value of proteomic analyses in providing mechanistic insights underlying disease progression.

## Materials and methods

### Differentiation of iPSC cultures

The use of human pluripotent stem cells was approved by the University of California, San Diego Institutional Review Board and Embryonic Stem Cell Research Oversight Committee. The generation and characterization of the WT83, Q83X, WT126, and N126I iPSC clonal lines was described and published in a separate study[11]. Human iPSC-derived forebrain NPCs were differentiated as previously described[19]. Forebrain NPCs were maintained at high density, grown on poly-ornithine/laminin-coated plates in NPC medium (DMEM-F12, 0.5% N2 and 1% B27 supplements (Life Technologies), 10 ng/mL EGF, 10 ng/mL FGF2) and passaged with Accutase.

### RA treatment and neuronal differentiation

On Day 0 of differentiation, forebrain NPCs were replated at 30,000–40,000 cells per cm^2^ in NPC medium without EGF/FGF2 and supplemented with 5μM Y-27632 (Stemgent) and 1μM retinoic acid (Tocris). Y-27632 was withdrawn on Day 3, and retinoic acid was withdrawn on Day 7. Starting on Day 3, the medium was supplemented with 200 μM ascorbic acid (Sigma), 1μM dibutyryl-cAMP (Sigma), 20 ng/mL BDNF (Life Technologies), and 20 ng/mL GDNF (Life Technologies) until Day 10, after which basal NPC medium minus EGF/FGF2 was used. Medium was partially changed every other day until Day 21 or Day 35 for downstream experiments. All NPCs used for RA treatment were passages 2–6.

### LIN28 overexpression and knockdown experiments

For the LIN28 overexpresssion experiments, we used a commercially available lentivirus expressing human LIN28 (Stemgent, #ST070016) and we used the uninfected cultures as "Control". 1 x 10^6^ transducing units (TU) of the LIN28 lentivirus were used to infect 200,000 WT83 NPCs at passage 4. For the LIN28 knockdown experiments, we used NPCs at passages 2–3 and utilized an shRNA construct targeting human LIN28 in the pLKO.1 vector (TRCN0000102579; Open Biosystems). As the "Control", a pLKO.1 vector containing an shRNA toward GFP was used (Open Biosystems). Both constructs were gifts from Dr. Eugene Yeo used in a previous publication[56]. The optimal titers of lentiviral supernatants were determined empirically and used to infect WT83 and Q83X NPCs.

### SILAC metabolic labeling, cell lysis, and sample processing

To generate near completely labeled iPSC-derived NPCs with stable isotope-labeled amino acids, cells were seeded in duplicate 10-cm plates at passages 2–3 and cultured for 12 days (~8 population doublings) in NPC media formulated with Arginine- and Lysine-depleted DMEM-F12 (Life Technologies) supplemented at a final concentration of 100 mg/L either with regular “light” L-Arg and L-Lys (Life Technologies) or “heavy” isotope-enriched [U-^13^C_6_,^15^N_2_]-L-Arg and [U-^13^C_6_,^15^N_4_]-L-Lys (Cambridge Isotopes). For SILAC of RA-differentiated cultures, NPC cultures at passages 2–3 (sister cultures of the NPC SILAC experiments) were re-seeded in duplicate 10-cm plates and SILAC labeled for two days in NPC medium prior to RA-treatment and harvested 21 days after the first day of RA treatment. Cell cultures were washed in cold PBS twice and then lysed in RIPA buffer (25mM Tris-HCl (pH 7.6), 150mM NaCl, 1% NP-40, 1% sodium deoxycholate, 0.1% SDS) for 1–2 hours directly on the culture dish while on ice. Protein extracts were collected with cell scrapers and the protein concentration was determined by BCA assay. Heavy labeled control extracts were then added 1:1 to light labeled RTT or control lysates and precipitated with methanol / chloroform.

### Mass spectrometry

For whole proteome analysis by MudPIT (LCLC-MS/MS), each analysis of 100 μg of total protein extract was processed to peptides as previously described[26, 57]. Each experiment was analyzed in 7–10 replicates, for a total of 154–220 hours of instrument time per experiment. Most of the data were acquired on a LTQ Velos Orbitrap mass spectrometer (Thermo Finnigan) with additional analysis on a LTQ Velos Orbitrap Elite and LTQ Orbitrap XL as previously described[58]. For LTQ Velos Orbitrap analysis, a cycle of one full-scan mass spectrum (400–1,800 m/z) at a resolution of 60,000, followed by 15 data-dependent MS/MS spectra at a 35% normalized collision energy was repeated continuously throughout each step of the multidimensional separation. For LTQ Orbitrap XL analysis, full-scan mass spectrum (400–1,600 m/z) at a resolution of 60,000 was followed by 9 data-dependent MS/MS spectra.

### Proteomic data analysis

Proteomic analyses (protein identification and quantification) were performed with Integrated Proteomics Pipeline—IP2 (Integrated Proteomics Applications, Inc., www.integratedproteomics.com) using ProLucid, DTASelect2, Census, and QuantCompare[33, 58, 59]. MS/MS spectra were searched with the in-house software ProLucid, against the EBI human IPI database (ftp://ftp.ebi.ac.uk/pub/databases/IPI, released in March 2007) concatenated to a decoy database in which the sequence for each entry in the original database was reversed. Peptides were required to posess at least one tryptic termini and be within 10PPM of the expected m/z. The resulting spectral matches were assembled and filtered using DTASelect with a protein false discovery rate of 1% for each analysis. Peptides that passed the filter were quantified using the in-house-developed software Census. A detailed description of the entire analysis workflow has been previously described[58]. In the current analysis, protein ratios and statistics were generated after grouping of all the quantified peptide ratios from all the replicate analysis. To control for multiple hypothesis testing, we used the Benjamini-Hochberg procedure[60] to estimate the false discovery rate (FDR) at the *t*-test *p*-value threshold of 0.05 for each proteomics experiment. To complete our inclusion criteria, a fold change threshold was then applied in addition to the *p*-value threshold to reduce false positives and therefore maximize specificity. All the proteomic data reported in this paper is fully available at ftp://massive.ucsd.edu/MSV000083090.

SILAC labeling efficiency was determined as previously described[59]. Briefly, analysis of 100 μg of the heavy labeled samples was performed by LCLC-MS/MS with Orbitraps mass spectrometers. Data was processed with SEQUEST, DTASelect, and Census software. We filtered our searches at 1% protein FDR based on target-decoy and to ensure confident quantitation we required peptide pairs to have a profile score of >0.5 in Census. We then calculated and compared the LIGHT / HEAVY peak areas and graphed the binned ratios. The labeling efficiency is reported as the average peptide ratio (light / heavy) after log transformation. Both paradigms involve determining ratios of “light” to “heavy” proteins towards the eventual calculation of the Mutant / WT ratio. In the Ratio of Ratios (RoR) analysis (mutant-“light” / wild type-“heavy” // wild type-“light” / wild type-“heavy”) paradigm, quantified proteins are normalized using a common internal standard which can accurately correct for incomplete labeling and other instrument-based biases[31]. In the Label Swap (LS) paradigm, we generate two ratios for each protein from four samples (mutant-“light” / wild type-“heavy” and wild type-“light” / mutant-“heavy”)[32, 33].

### Statistical analysis of SILAC results

Let Pr[*Q*_*L*_,*N*_*R*_,*Q*_*R*_,*N*_*L*_] be the joint probability of identifying a protein as being downregulated using all of our inclusion criteria in both Q83X and N126I neural cultures by chance. More precisely, *Q*_*L*_ corresponds to the event of observing a protein meeting our inclusion criteria for the LS paradigm with the Q83X neural culture. *N*_*L*_ represents the same event but in the N126I neural culture. *N*_*R*_ and *Q*_*R*_ consist of the events of a protein to be down-regulated according to the inclusion criteria of the RoR paradigm in the N126I and Q83X cultures respectively. These events share dependencies because of their occasional usage of the same cell lines and similar analytical paradigms. In order to compute Pr[*Q*_*L*_,*N*_*R*_,*Q*_*R*_,*N*_*L*_], these dependencies have to be accounted for. We therefore estimated this probability using chain rule,

$$Pr[Q_{L},N_{R},Q_{R},N_{L}]=\mathrm{Pr}\left[Q_{L}\right]∙\mathrm{Pr}\left[N_{R}|Q_{L}\right]∙\mathrm{Pr}\left[Q_{R}|Q_{L},N_{R}\right]∙\mathrm{Pr}\left[N_{L}|Q_{L},N_{R},Q_{R}\right]$$

Pr[*Q*_*L*_] is inferred from the data using the fraction of proteins passing the inclusion criteria over all significant proteins (*p*-value <0.05) from the LS analysis of the Q83X cells. An estimate of Pr[*N*_*R*_|*Q*_*L*_] is computed using Bayes’ rule:

$$\mathrm{Pr}\left[N_{R}|Q_{L}\right]=\frac{\mathrm{Pr}\left[N_{R}\right]∙Pr[Q_{L}|N_{R}]}{\zeta }$$

where Pr[*Q*_*L*_|*N*_*R*_] is estimated by the number of proteins meeting both inclusion criteria of LS in Q83X cells and RoR in N126I cells over the number of proteins passing the criteria of the former. *ζ* is a normalization factor and Pr[*N*_*R*_] is estimated using the same strategy as Pr[*Q*_*L*_].

It is unlikely that we can exactly compute Pr[*Q*_*R*_|*Q*_*L*_,*N*_*L*_]. Nevertheless, Bayes’ rule can be used to estimate this probability by assuming the conditional independence of *Q*_*L*_ and *N*_*R*_ given *Q*_*R*_:

$$\mathrm{Pr}\left[Q_{R}|Q_{L},N_{R}\right]=\frac{\mathrm{Pr}\left[Q_{R}\right]∙\mathrm{Pr}\left[Q_{L}|Q_{R}\right]∙Pr[N_{R}|Q_{R}]}{\zeta }$$

Probabilities on the right hand side of the equation are inferred from the data using the strategy detailed previously. As for Pr[*N*_*L*_|*Q*_*L*_,*N*_*R*_,*Q*_*R*_], using Bayes’ rule and the conditional independence of *N*_*R*_ and *Q*_*L*_, and *N*_*R*_ and *Q*_*R*_ given *N*_*L*_,

$$\mathrm{Pr}\left[N_{L}|Q_{L},N_{R},Q_{R}\right]=\frac{\mathrm{Pr}\left[N_{L}\right]∙\mathrm{Pr}[N_{R}|N_{L}]∙Pr[Q_{L},Q_{R}|N_{L}]}{\zeta }$$

Pr[*N*_*L*_] and Pr[*N*_*R*_|*N*_*L*_] are estimated from the data. However, since *Q*_*L*_ and *Q*_*R*_ involve the same cell line, they are likely to not be conditionally independent given *N*_*L*_. Hence, using chain rule,

$$\mathrm{Pr}\left[Q_{L},Q_{R}|N_{L}\right]=\mathrm{Pr}\left[Q_{L}|N_{L}\right]∙\mathrm{Pr}\left[Q_{R}|Q_{L},N_{L}\right]$$

where Pr[*Q*_*L*_|*N*_*L*_] is inferred as shown previously. Finally, using Bayes’ rule and assuming the conditional independence of *Q*_*L*_ and *N*_*L*_ given *Q*_*R*_,

$$\mathrm{Pr}\left[Q_{R}|Q_{L},N_{L}\right]=\frac{\mathrm{Pr}\left[Q_{R}\right]∙\mathrm{Pr}\left[Q_{L}|Q_{R}\right]∙Pr[N_{L}|Q_{R}]}{\zeta }$$

where Pr[*N*_*L*_|*Q*_*R*_] is estimated from the data.

### IRFL-based Odyssey Western blotting & In-Cell Western

For IRFL-based Odyssey Western blots, cultures were lysed with RIPA buffer and protein lysates were collected using standard methods. For each sample, 10 μg of total proteins were loaded and run per well in NuPAGE gels (10% or 4–12% gradient gel; Invitrogen). The gels were subsequently transferred on to low-fluorescence PVDF membrane (Invitrogen). Membranes containing protein bands were blocked in Li-COR Odyssey Blocking buffer (Li-COR Biosciences) for 1.5 hr at room temperature, washed 5x with PBS + 0.1% Tween solution (PBST), and incubated in Odyssey Blocking Buffer containing primary antibodies (Mouse anti-SMI21R, Covance; Chicken anti-MAP2, Abcam; Rabbit anti-Lin28A, Cell Signaling; Mouse anti-beta-actin, Li-COR Biosciences) for 4 hrs at room temperature, or overnight at 4ºC. Membranes were then washed 5 times with PBST, and incubated in Odyssey Blocking Buffer containing secondary antibodies for IRFL detection (Goat anti-Mouse 680/800, Goat anti-Rabbit 680/800, all from Li-COR Biosciences) for 30 mins at room temperature. 0.01% SDS was added to reduce background fluorescence. Subsequently, membranes were washed 3 times with PBST and rinsed with PBS. Wet membranes were placed on Li-COR Odyssey detection machine to scan and record fluorescence signals. Protein band intensities were recorded as fluorescence intensity using Li-COR image analysis software. Bands for proteins of interest were normalized over beta-actin (control) band intensities on the same blot.

For IRFL-Based In-Cell Western procedures, NPCs or iPSC-derived neuronal cultures grown on glass coverslips were fixed with 4% PFA + 20% sucrose solution at room temperature for 15 min. Cultures were then rinsed 3 times with PBS and stored in PBS + 0.1% Na-azide at 4ºC until antibody staining. Prior to staining, cultures were permeabilized 5 times for 5 mins with PBS + 3.3% BSA + 0.1% Triton solution on a gentle shaker. Cultures were then rinsed with PBST and blocked for 1.5 hrs at room temperature, or overnight at 4ºC, in Odyssey Blocking Buffer. Primary and secondary antibodies were diluted using Odyssey Blocking Buffer containing 0.01% SDS. Primary antibodies used include: Mouse anti-SMI21R, Covance; Chicken anti-MAP2, Abcam; Rabbit anti-synapsin, Cell Signaling Technology; Rabbit anti-vGlut1, Abcam; Mouse anti-VGAT, Cell Signaling technology. Secondary antibodies include: Goat anti-Mouse 680/800, Goat anti-Rabbit 680/800, all from Li-COR Biosciences. Cultures were incubated in primary antibodies were incubated for at least 4 hrs at room temperature, or overnight at 4ºC; in secondary antibodies for 30 minutes to 1 hr at room temperature.

For imaging, glass coverslips were retrieved from the holder wells and placed facedown on the Odyssey imaging platform for optimal focal plane position during scanning. Images were analyzed using the Li-COR analysis software. Whole areas of the coverslips were included in total fluorescence detection. Signals were normalized over beta-actin (control) channel.

### Genome editing of hESCs

A *MECP2* knockout model was made using the commercially available male human embryonic stem cell (hESC) line, SA001 (Cellartis). To knockout the MECP2 gene we used a plasmid-based antibiotic selection cassette (loxP-PGK-gb2-neo-loxP plasmid from GeneBridges), together with zinc finger nucleases (ZFNs) designed for the target (Sigma Aldrich product # CKOZFND-1505-1KT). For the “WT” control line, we used the same SA001 line with the targeting vector used to generate the MECP2 knockout inserted into a safe harbor locus, AAVS1, using ZFNs targeted to this site. A similar procedure, as described below for the generation of the MECP2 lines, was followed for generating the WT lines. Nucleofection of ZFNs and targeting plasmid was done in 100μL cuvettes using the Amaxa 4D-Nucleofector X Unit (Lonza), program DN-100. For each nucleofection, 1μg each of the two ZFNs and 1μg of the targeting plasmid was used.

Briefly, SA001 cells (low passage, karyotypically normal) were grown on Matrigel-coated 10-cm plates in mTeSR1 medium (StemCell Technologies) until ~80% confluenecy. Cells were dissociated with Accutase, resuspended in 10mL mTeSR1, and cell count was determined. 4 million cells per nucleofection was added to a sterile 1.5 mL Eppendorf and briefly pelleted. After removing all supernatant, the cell pellet was resuspended in 200 μL P3 solution (Lonza) and DNA for two transfections was added to the cell suspension. 100 μL of the cell suspension plus DNA was transferred to two cuvettes and nucleofected immediately. Nucleofected cells were then transferred to a 10-cm plate containing 12 mL mTeSR1 and 10 μM ROCK inhibitor.

One day post-nucleofection, the media was changed to mTeSR1 only. Every day thereafter, until colonies were formed, the media was changed to mTeSR1 plus 0.2 mg/mL G-418. 11–13 days post-nucleofection, when the colonies were 1–2 mm in diameter, colonies were manually selected and transferred to a 48-well Matrigel plate with mTeSR1 + 10μM ROCK inhibitor. After cells attached (1–2 hours), media was changed to mTeSR1 only. Daily media changes were performed as before, until most wells were 80–90% confluent.

For selection of clones, all lines were screened by PCR for target vector insertion at the appropriate site. For the MECP2 knockout we were also able to perform screening by immunofluorescence, which allowed us to easily detect heterogeneous populations (S6A Fig). For IF screening we used a rabbit anti-MECP2 antibody (Cell Signalling D4F3) and imaged with the Operetta System (Perkin Elmer).

The first round of colony selection resulted in a few clones with predominantly knockout cells but still heterozygous for some non-targeted cells. Therefore, we had to do a second round of selection. We were able to achieve homogeneous clonal lines by splitting the cultures at very low density (800 cells/cm^2^) and manually selecting sub-colonies. As a final validation, homozygous MECP2 knockout clones were screened for number of target vector insertions by digital droplet PCR (ddPCR) to exclude the possibility of additional spurious insertions (S6B and S6C Fig).

### Establishment of stable NPCs and serial passaging

Briefly, human pluripotent stem cells (hPSCs) were dissociated to single cells and transferred into AggreWell-800 plates for formation of aggregates with defined cell numbers. Neural aggregates were formed in media containing 50% DMEM/F12 with Glutamax I (Invitrogen), 50% Neurobasal (Invitrogen), B27 supplement without vitamin A (Invitrogen), N2 supplement (Invitrogen), and 0.1% beta-mercaptoethanol (N2B27) supplemented with FGF-2 (5ng/mL), Noggin (266ng/mL), SB 431542 (20μM). The application of Noggin and SB-431542 (‘dual SMAD’) induced neuralization[61]. Each day, a partial media change was performed. After five days of neutralization, neural aggregates were plated onto poly-ornithine/laminin-coated plates and allowed to form neural rosettes under continued dual SMAD conditions. After approximately 4 days, neural rosettes were selectively isolated using STEMdiff Neural Rosette Selection Medium (StemCell Technologies), replated onto poly-ornithine/laminin-coated plates and expanded under dual SMAD conditions. Cultures were then trypsinized, replated onto poly-ornithine/laminin-coated plates and cultured in N2B27 supplemented with FGF-2, EGF, and BDNF (FEB) at high cell density. Continued passaging in FEB with step-wise reduction in seeding density produced a stable neural stem/progenitor cell line.

NPC cell lines were thawed from liquid nitrogen storage. Samples were collected for Western analysis from the first, third and fifth passage after thawing. The corresponding passage number of the relevant cell lines are as follows: WT83c7 NPC (passage 9, 11, 13); Q83Xc1 NPC (passage 10, 12, 14); Q83Xc3 NPC (passage 10, 12, 14); SA001_MECP2GEc1 NPC (passage 24, 26, 28); SA001_CtrlGEc16B2 NPC (passage 24,26,28). For each passage, four biological replicates were performed per cell line. Cells were seeded at a density of 25,000/cm^2^ on 12-well plates, collected in 100 μL Laemmli/DTT buffer, and stored at -20°C. Samples were run on a 4–12% Bis-Tris gel. Due to the number of samples, in order to compare all data points, we made a pooled sample, which was run in three lanes on each gel and used for normalization. The blots were probed as described above. Each data point was first normalized to actin and then to the normalized pooled sample.

### MECP2 Chromatin Immunoprecipitation (ChIP)

Passage 31 WT83 iPSCs and passage 7 NPCs were grown to confluence in 10-cm plates and harvested for ChIP. The ChIP-IT Express Enzymatic kit (Active Motif) was used according to manufacturer’s instructions, with the addition of a DNA clean-up step using phenol:chloroform:isoamyl alcohol at the end point. Briefly, cells were fixed and lysed with 20 strokes of a dounce homogenizer to aid the release of nuclei. The cellular extract was incubated with enzymatic shearing cocktail solution for 10 minutes at 37°C, and mixed every two minutes to increase shearing efficiency. Each sample was incubated with 2 ng of MECP2 antibody (Diagenode), or with controls, one with RNA pol II (RNAP) or IgG for positive or negative controls, respectively. Primers used for the LIN28 promoter region were: forward GAGCTGGGAATCAAGACAGC and reverse GAGTTGAACGCTCTGGCTTC; primer sequences for the BDNF promoter were: forward AAGCATGCAATGCCCTGGAA and reverse TGCCTTGACGTGCGCTGTCAT. Real-time PCR reaction was performed using the iQ SYBR green reagent (BioRad), using a CFX_2stepAmp standard protocol with melting curve step added: 95°C for 10 minutes; 40 cycles of 95°C for 15 seconds and 60°C for 60 seconds; 95°C for 10 seconds, 65°C for 5 seconds and final 95°C for 5 seconds for the melting curve.

## Supporting information

S1 Fig Characterization of 3-week-old RA-differentiated cultures from WT83, Q83X, WT126, and N126I NPCs. A. Comparison of MAP2+ and GFAP+ cells in WT83 and Q83X cultures. Flow cytometry gating parameters using isotype control antibodies are shown. The lower four panels are the same as in Fig 1D.
B. Comparison of MAP2+ and GFAP+ cells in WT126 and N126I cultures. Flow cytometry gating parameters using isotype control antibodies are shown.
(PDF)

S2 Fig SILAC labeling of RA-differentiated cultures from WT83, Q83X, WT126, and N126I NPCs. A. WT83, Q83X, WT126, and N126I 3-week-old cultures in SILAC medium with either “Light” (upper panels) or “Heavy” (lower panels) amino acids. Scale bar is 200 μm.
B. Graphs of peptide frequency versus light/heavy ratio for the indicated RA-treated differentiated cultures and biological replicates. Also indicated is the average peptide labeling efficiency for each replicate.
C. List of neuron-, astrocyte- and oligodendrocyte-specific proteins mapped to genes in the RoR and LS datasets. Red numbers indicate upregulation and blue downregulation. Proteins with significant *p*-values are highlighted in yellow.
(PDF)

S3 Fig Characterization of undifferentiated NPCs derived from WT83 and Q83X iPSCs. A. Flow cytometry data showing expression of CD271/p75 (upper plots) and CD56/NCAM (lower plots) in WT83 NPCs at passage 5 and two Q83X NPC clones each at passage 6. Percentage positive populations in Quadrant 1 are summarized in a table (bottom right corner). NCAM is a neuroectoderm marker and p75 is an early neural crest marker.
B. WT83, Q83X, WT126, and N126I NPCs cultured in SILAC medium with either “Light” (upper panels) or “Heavy” (lower panels) amino acids. Scale bar is 200 μm.
C. Graphs of peptide frequency versus light/heavy ratio for the indicated NPC cultures and biological replicates. Also indicated is the average peptide labeling efficiency for each replicate.
D. Western blot of LIN28 expression in WT83 and Q83X NPCs at passage 4 (P4) and passage 8 (P8).
E. Representative Odyssey Western blot of LIN28 expression in WT83 and two clones of Q83X NPCs quantified in Fig 3F. Blot shows NPC samples from the 3^rd^ passage after thaw. Four biological replicates are shown per sample.
(PDF)

S4 Fig MECP2 interacts with the promoter region of *LIN28*. ChIP-qPCR using MECP2 antibodies show a significant enrichment fold in LIN28 promoter-specific amplification compared to IgG in both WT iPSCs (A) and NPCs (B). This is also seen with primers specific for BDNF, a gene that is known to be regulated by MECP2. Positive control antibodies for RNA pol II (RNAP) show the efficiency of the ChIP reaction for each primer set.
(PDF)

S5 Fig Many perturbed proteins are astrocyte markers previously profiled in human cortical spheroid cultures derived from iPSCs[35]. A. Proteins that overlap with Early Pseudotime Markers.
B. Proteins that overlap with Middle Pseudotime Markers.
C. Proteins that overlap with Late Pseudotime Markers.
D. Proteins that overlap with Mature Astrocyte Markers.
(PDF)

S6 Fig Generation of ZFN-mediated MECP2 KO hESCs. A. Immunofluorescence staining for MECP2 (in green) before ZFN-mediated KO (left panel), after ZFN delivery (middle panel), and after clonal selection (right panel).
B. ddPCR graph showing single Neo cassette integration in MECP2^-/y^ hESC clones.
C. ddPCR data quantification showing Neo cassette copy numbers per haploid genome and associated data.
D. Immunofluorescence staining for MECP2 (red) and Nestin (green) in WT vs MECP2^-/y^ hESC-derived NPCs.
(PDF)

S1 Table Intersectional list of perturbed proteins with known cell-type specific markers. Spreadsheet of proteins identified in our SILAC MS data shown in S2C Fig and S5 Fig that overlap with previously published gene lists.
(XLSX)
