# Supplementary figures and images for: Proteomic analyses reveal misregulation of LIN28 expression and delayed timing of glial differentiation in human iPS cells with MECP2 loss-of-function
Source: PLoS One. 2019 Feb 21;14(2):e0212553. doi: 10.1371/journal.pone.0212553 (PMC6383942; doi:10.1371/journal.pone.0212553)

**A**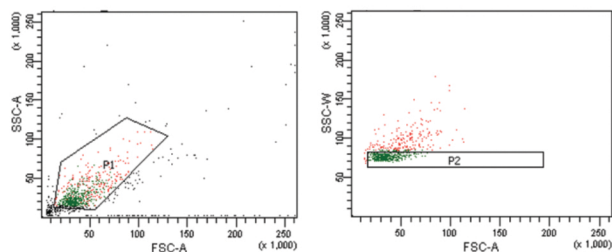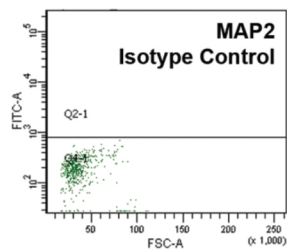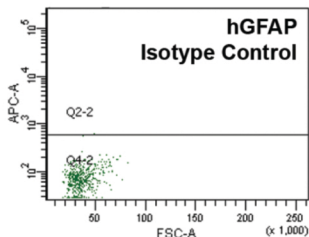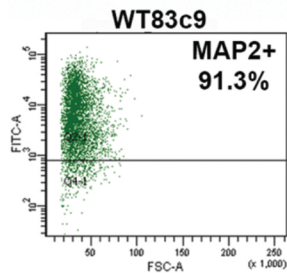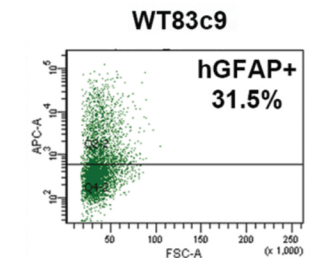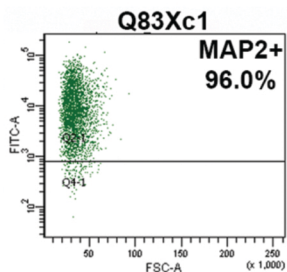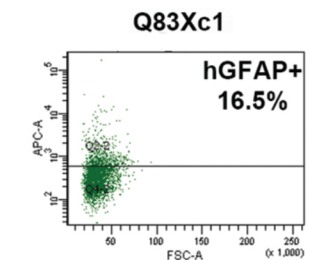**B**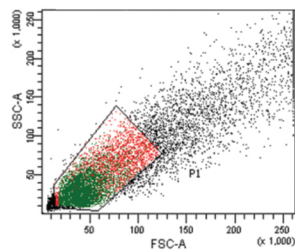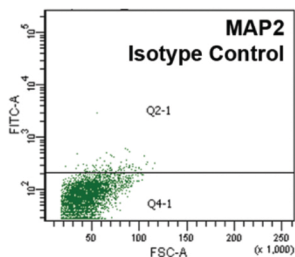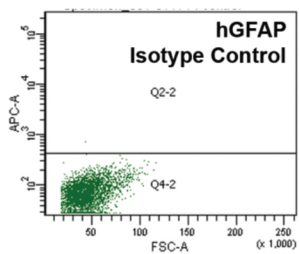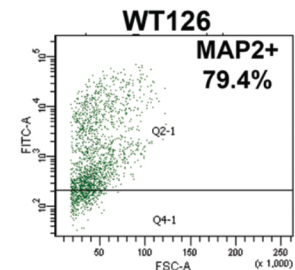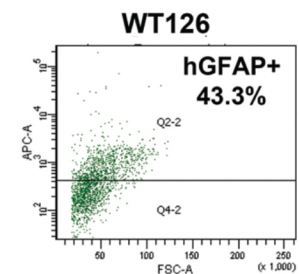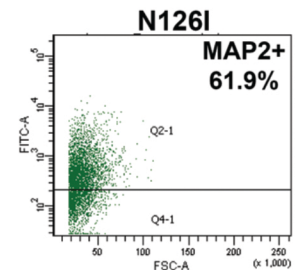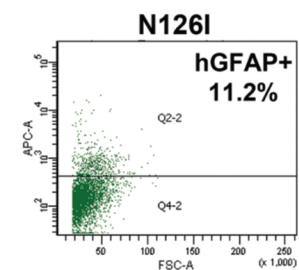

Supplement: S1 Fig — A. Comparison of MAP2+ and GFAP+ cells in WT83 and Q83X cultures. Flow cytometry gating parameters using isotype control antibodies are shown. The lower four panels are the same as in Fig 1D. B. Comparison of MAP2+ and GFAP+ cells in WT126 and N126I cultures. Flow cytometry gating parameters using isotype control antibodies are shown. (PDF) [file pone.0212553.s001.pdf]

**A**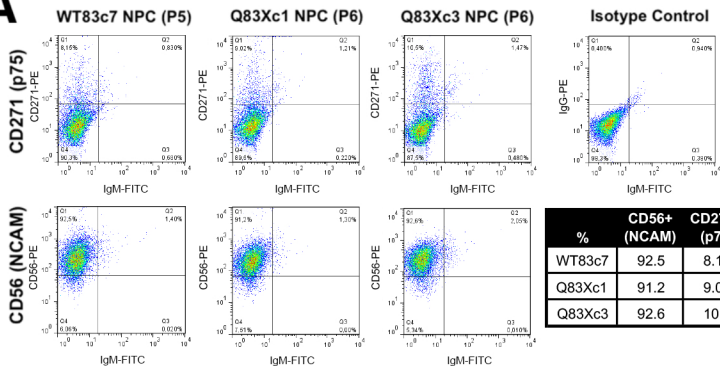**B**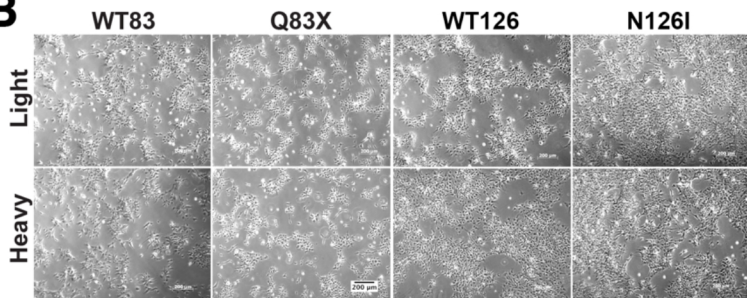**C**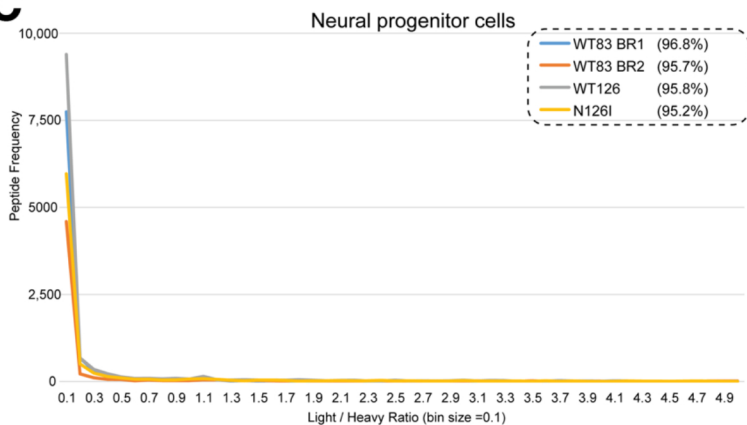**D**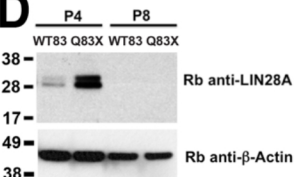**E**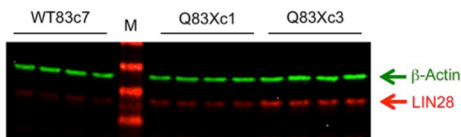

Supplement: S3 Fig — A. Flow cytometry data showing expression of CD271/p75 (upper plots) and CD56/NCAM (lower plots) in WT83 NPCs at passage 5 and two Q83X NPC clones each at passage 6. Percentage positive populations in Quadrant 1 are summarized in a table (bottom right corner). NCAM is a neuroectoderm marker and p75 is an early neural crest marker. B. WT83, Q83X, WT126, and N126I NPCs cultured in SILAC medium with either “Light” (upper panels) or “Heavy” (lower panels) amino acids. Scale bar is 200 μm. C. Graphs of peptide frequency versus light/heavy ratio for the indicated NPC cultures and biological replicates. Also indicated is the average peptide labeling efficiency for each replicate. D. Western blot of LIN28 expression in WT83 and Q83X NPCs at passage 4 (P4) and passage 8 (P8). E. Representative Odyssey Western blot of LIN28 expression in WT83 and two clones of Q83X NPCs quantified in Fig 3F. Blot shows NPC samples from the 3rd passage after thaw. Four biological replicates are shown per sample. (PDF) [file pone.0212553.s003.pdf]
