# Supplementary material for: Proteomic analyses reveal misregulation of LIN28 expression and delayed timing of glial differentiation in human iPS cells with MECP2 loss-of-function
Source: PLoS One. 2019 Feb 21;14(2):e0212553. doi: 10.1371/journal.pone.0212553 (PMC6383942; doi:10.1371/journal.pone.0212553)
Supplement: S2 Fig — A. WT83, Q83X, WT126, and N126I 3-week-old cultures in SILAC medium with either “Light” (upper panels) or “Heavy” (lower panels) amino acids. Scale bar is 200 μm. B. Graphs of peptide frequency versus light/heavy ratio for the indicated RA-treated differentiated cultures and biological replicates. Also indicated is the average peptide labeling efficiency for each replicate. C. List of neuron-, astrocyte- and oligodendrocyte-specific proteins mapped to genes in the RoR and LS datasets. Red numbers indicate upregulation and blue downregulation. Proteins with significant p-values are highlighted in yellow. (PDF) [file pone.0212553.s002.pdf]

A

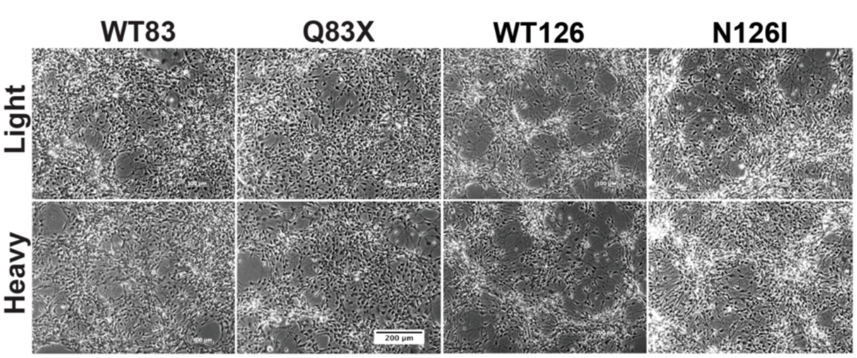

B

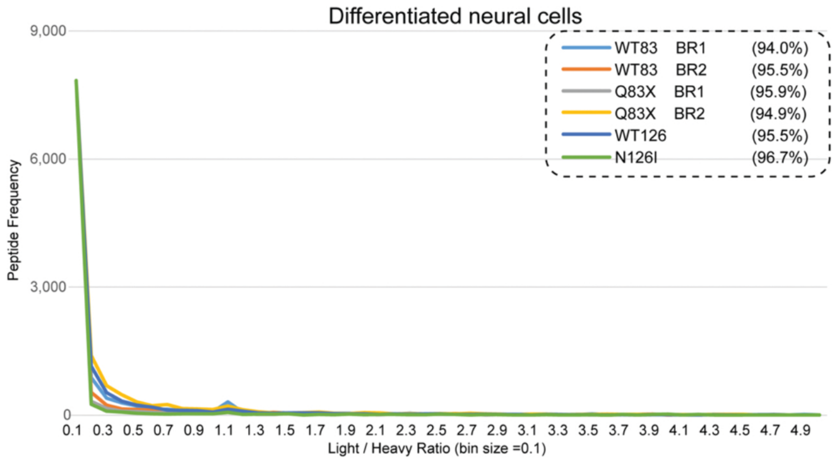

C

Proteins mapped to mouse neuron-specific genes

	**Ratio of Ratios (RoR)**				**Label Swap (LS)**			
	Q83X		N126I		Q83X		N126I	
Gene	Ratio	*p*-value	Ratio	*p*-value	Ratio	*p*-value	Ratio	*p*-value
Odz2	0.032	0.912	NA	X	**-0.168**	**0.039**	**1.496**	**0.000**
Myt1l	NA	X	NA	X	0.888	0.203	0.722	X
Gabrg2	NA	X			-0.059	0.412		
Plcxd3	-1.411	X	0.104	X	-1.286	X	0.202	X
**Snap25**	**-0.901**	**0.000**	**-0.263**	**0.007**	**-0.737**	**0.000**	**-0.136**	**0.005**
Pgm2l1	**-0.604**	**0.003**	**0.317**	**0.000**	**-0.396**	**0.000**	0.202	0.013
**Stmn2**	**-0.775**	**0.000**	**-0.200**	**0.024**	-0.234	0.062	**-0.120**	**0.053**
Prdm8			NA	X	1.251	X		
Glra2	-0.501	X	0.086	0.560	-0.556	0.060	0.263	0.297
**Calb1**	-0.274	0.080	-0.207	0.618	**-1.059**	**0.001**	-0.120	0.342
**Syt1**	**-0.488**	**0.000**	**-0.246**	**0.000**	**-0.246**	**0.000**	**-0.105**	**0.001**
Scg2	**-0.931**	**0.000**	**0.317**	**0.000**	**-0.340**	**0.000**	**0.401**	**0.000**

Proteins mapped to mouse astrocyte-specific genes

	**Ratio of Ratios (RoR)**				**Label Swap (LS)**			
	Q83X		N126I		Q83X		N126I	
Gene	Ratio	*p*-value	Ratio	*p*-value	Ratio	*p*-value	Ratio	*p*-value
**S100b**	**-1.280**	**0.000**	**-1.399**	**0.000**	**-0.786**	**0.000**	**-1.415**	**0.000**
Aldoc	**-1.021**	**0.000**	0.132	0.106	**-0.535**	**0.000**	-0.029	0.154
**Gfap**	**-3.489**	**0.034**	**-0.924**	**0.000**	**-2.943**	**0.001**	**-1.218**	**0.000**
Slc1a3	1.258	0.341	-0.135	0.188	1.245	0.159	**-0.234**	**0.000**
**Atp1a2**	**-0.749**	**0.001**	**-0.332**	**0.000**	**-0.535**	**0.006**	**-0.152**	**0.000**
Prodh			NA	X			**0.934**	**0.001**
Slc1a2	-0.658	0.081	1.072	0.098	**-0.322**	**0.000**	0.070	0.868

Proteins mapped to mouse oligodendrocyte-specific genes

	**Ratio of Ratios (RoR)**				**Label Swap (LS)**			
	Q83X		N126I		Q83X		N126I	
Gene	Ratio	*p*-value	Ratio	*p*-value	Ratio	*p*-value	Ratio	*p*-value
Plp1	NA	X	-1.127	X	0.379	0.316	-0.621	X

Ratio	Log(2)
0.25	-2.000
0.5	-1.000
0.8	-0.322
0.9	-0.152
1	0.000
1.1	0.138
1.2	0.263
2	1.000
4	2.000
