# Supplementary material for: Proteomic analyses reveal misregulation of LIN28 expression and delayed timing of glial differentiation in human iPS cells with MECP2 loss-of-function
Source: PLoS One. 2019 Feb 21;14(2):e0212553. doi: 10.1371/journal.pone.0212553 (PMC6383942; doi:10.1371/journal.pone.0212553)
Supplement: S4 Fig — ChIP-qPCR using MECP2 antibodies show a significant enrichment fold in LIN28 promoter-specific amplification compared to IgG in both WT iPSCs (A) and NPCs (B). This is also seen with primers specific for BDNF, a gene that is known to be regulated by MECP2. Positive control antibodies for RNA pol II (RNAP) show the efficiency of the ChIP reaction for each primer set. (PDF) [file pone.0212553.s004.pdf]

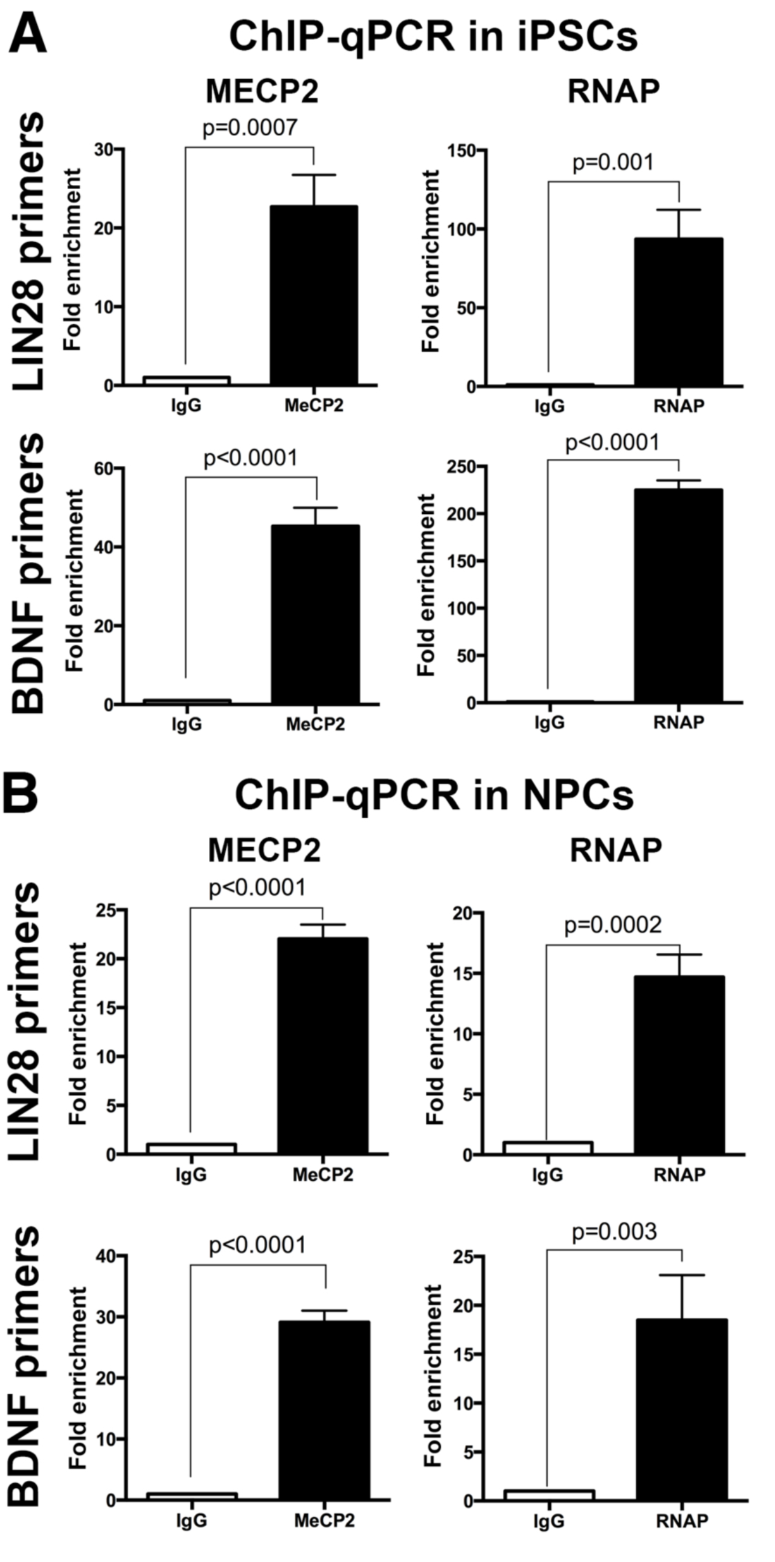
A
ChIP-qPCR in iPSCs
MECP2
RNAP
LIN28 primers
BDNF primers
Fold enrichment
p=0.0007
p=0.001
p<0.0001
p<0.0001
IgG
MeCP2
RNAP
B
ChIP-qPCR in NPCs
MECP2
RNAP
LIN28 primers
BDNF primers
p<0.0001
p=0.0002
p<0.0001
p=0.003
