# Supplementary material for: Proteomic analyses reveal misregulation of LIN28 expression and delayed timing of glial differentiation in human iPS cells with MECP2 loss-of-function
Source: PLoS One. 2019 Feb 21;14(2):e0212553. doi: 10.1371/journal.pone.0212553 (PMC6383942; doi:10.1371/journal.pone.0212553)
Supplement: S5 Fig — A. Proteins that overlap with Early Pseudotime Markers. B. Proteins that overlap with Middle Pseudotime Markers. C. Proteins that overlap with Late Pseudotime Markers. D. Proteins that overlap with Mature Astrocyte Markers. (PDF) [file pone.0212553.s005.pdf]

A

Early Pseudotime Markers from Sloan et al. (2017)
Cluster 1 (enriched at early pseutotimes)

	Ratio of Ratios (RoR)				Label Swap (LS)			
	Q83X		N126I		Q83X		N126I	
Gene	Ratio	*p*-value	Ratio	*p*-value	Ratio	*p*-value	Ratio	*p*-value
PLS3	-1.442	0.052	-0.767	0.096	0.029	0.654	**-0.556**	**0.000**
**ANXA2**	**-0.301**	**0.000**	**-0.181**	**0.044**	**-0.515**	**0.000**	**-0.120**	**0.001**
CTSC	**0.356**	**0.000**	**0.451**	**0.000**	0.000	0.548	**0.333**	**0.000**
EBF2			NA	X				
ACTG2	**-0.391**	**0.000**	0.014	0.574	**-0.252**	**0.000**	0.000	0.616
CNTNAP2	**-1.285**	**0.000**	**0.475**	**0.008**	**-0.535**	**0.000**	**0.401**	**0.002**
**DCX**	**-0.598**	**0.000**	**-0.097**	**0.000**	**-0.286**	**0.000**	-0.014	0.275
FBLN1	NA	X						
RRM2	NA	X	**-0.884**	**0.003**	-0.515	X	**-0.494**	**0.037**
**INA**	**-0.675**	**0.000**	**-0.056**	**0.001**	**-0.474**	**0.000**	**-0.059**	**0.000**
NCAPH	**1.209**	**0.012**	-0.354	0.208	0.566	0.109	**-0.474**	**0.000**
CRABP1	**0.475**	**0.000**	**0.132**	**0.000**	**0.251**	**0.000**	0.057	0.070
TIMELESS			NA	X				
**NEFL**	**-1.322**	**0.000**	**-0.284**	**0.000**	**-0.434**	**0.000**	**-0.105**	**0.000**
FAM105A			0.863	X			**-0.396**	**0.028**
GINS2	NA	X	NA	X	1.098	0.407	-0.494	0.230
UBE2C	NA	X			1.379	X	**-1.000**	**0.009**
PBK	1.510	X	-1.267	X	1.390	X	-0.889	X
GPR50	NA	X	-0.637	X	**0.799**	**0.000**	0.632	X
DNMT3B	NA	X			**0.287**	**0.002**		
ELAVL4	**0.366**	**0.006**	**-0.593**	**0.000**	**-0.089**	**0.045**	**-0.713**	**0.000**

B

Middle Pseudotime Markers from Sloan et al. (2017)
Cluster 2 (enriched at middle pseudotimes)

	Ratio of Ratios (RoR)				Label Swap (LS)			
	Q83X		N126I		Q83X		N126I	
Gene	Ratio	*p*-value	Ratio	*p*-value	Ratio	*p*-value	Ratio	*p*-value
SLC2A1	0.635	X	NA	X	-0.074	0.101	**-0.556**	**0.001**
**RTN1**	**-0.691**	**0.000**	**-0.475**	**0.000**	**-0.269**	**0.000**	**-0.474**	**0.000**
**ATP1A2**	**-0.749**	**0.001**	**-0.332**	**0.000**	**-0.535**	**0.006**	**-0.152**	**0.000**
C9orf126	0.068	X	0.903	X	**-0.201**	**0.004**	-0.136	0.074
DPH2	0.291	X	0.229	X	0.202	0.524	-0.152	0.284
CLU			-1.273	X	-0.252	X	**-1.089**	**0.012**
SLC1A3	1.258	0.341	-0.135	0.188	1.245	0.159	**-0.234**	**0.000**
ALDOC	**-1.021**	**0.000**	0.132	0.106	**-0.535**	**0.000**	-0.029	0.154
**AKR1C3**	-0.539	X	-0.103	0.410	-0.644	X	**-0.201**	**0.028**
NEK	Multiple	X	Multiple	X	Multiple	X	Multiple	X
PTN	0.282	0.462	0.663	0.335	0.604	0.203	1.239	0.137
NTRK2	-0.210	0.085	**0.181**	**0.020**	**0.566**	**0.038**	-0.014	0.409
SCUBE2	NA	X	-1.317	0.133	-0.120	0.294	**-0.811**	**0.002**
TNC	NA	X	**0.397**	**0.006**			**0.345**	**0.000**

C

Late Pseudotime Markers from Sloan et al. (2017)
Cluster 3 (enriched at late pseudotimes)

	Ratio of Ratios (RoR)				Label Swap (LS)			
	Q83X		N126I		Q83X		N126I	
Gene	Ratio	*p*-value	Ratio	*p*-value	Ratio	*p*-value	Ratio	*p*-value
PLTP			NA	X			-0.889	X
PPAP2B					-2.184	X		
**CHL1**	**-0.590**	**0.050**	-1.104	X	**-0.152**	**0.025**	-0.737	X
HTRA1	NA	X	**-0.841**	**0.023**	-0.152	X	**-1.252**	**0.001**
ENDOD1	0.303	0.218	0.089	0.693	**-0.168**	**0.007**	**0.263**	**0.002**
CSRP1	**0.621**	**0.007**	NA	X	**0.485**	**0.022**	**-0.644**	**0.000**
GPC5	NA	X			-1.184	X		
**GATM**	**-0.702**	**0.000**	-1.896	0.186	-0.252	0.063	**-0.862**	**0.000**
ACSS1			NA	X			-0.252	0.269
BCAN	-3.733	X	-0.453	0.103	0.163	0.787	**-0.322**	**0.038**
**VSNL1**	**-1.184**	**0.000**	-0.448	0.351	**-1.059**	**0.000**	0.138	0.804
GFRA1	NA	X	0.864	0.240	-0.184	0.310	0.566	0.124
DBC1	NA	X	NA	X	-0.786	X	-0.120	0.626
APOE					2.755	0.151		
MOXD1			NA	X	**-0.269**	**0.005**		
SLC1A2	-0.658	0.081	1.072	0.098	**-0.322**	**0.000**	0.070	0.868
**TPP1**	**-0.332**	**0.032**	**-0.278**	**0.002**	**-0.168**	**0.029**	**-0.286**	**0.000**
**GSN**	**-0.376**	**0.002**	**-0.341**	**0.000**	**-0.396**	**0.000**	**-0.474**	**0.000**

D

Mature Astrocyte Markers from Sloan et al. (2017)
Genes enriched in primary mature astrocytes / hCS astrocytes

	Ratio of Ratios (RoR)				Label Swap (LS)			
	Q83X		N126I		Q83X		N126I	
Gene	Ratio	*p*-value	Ratio	*p*-value	Ratio	*p*-value	Ratio	*p*-value
S100A13	-0.106	0.806			-0.120	0.286	-0.029	0.378
ALDH1A1					0.926	X		
CSRP1	**0.621**	**0.007**			**0.485**	**0.022**	**-0.644**	**0.000**
GSTM5			**-2.822**	**0.001**			**-2.322**	**0.010**
APOC1					-0.415	0.243		
CPNE6			-0.648	X			**-0.578**	**0.000**
UCHL5	-0.354	0.273	0.243	0.468	**-0.184**	**0.047**	-0.396	0.107
ME1	NA	X	**0.908**	**0.000**	**-0.599**	**0.033**	**0.465**	**0.000**
SLC1A2	-0.658	0.081	1.072	0.098	**-0.322**	**0.000**	0.070	0.868

Ratio	Log(2)
0.25	-2.000
0.5	-1.000
0.8	-0.322
0.9	-0.152
1	0.000
1.1	0.138
1.2	0.263
2	1.000
4	2.000
