# Supplementary material for: Proteomic analyses reveal misregulation of LIN28 expression and delayed timing of glial differentiation in human iPS cells with MECP2 loss-of-function
Source: PLoS One. 2019 Feb 21;14(2):e0212553. doi: 10.1371/journal.pone.0212553 (PMC6383942; doi:10.1371/journal.pone.0212553)

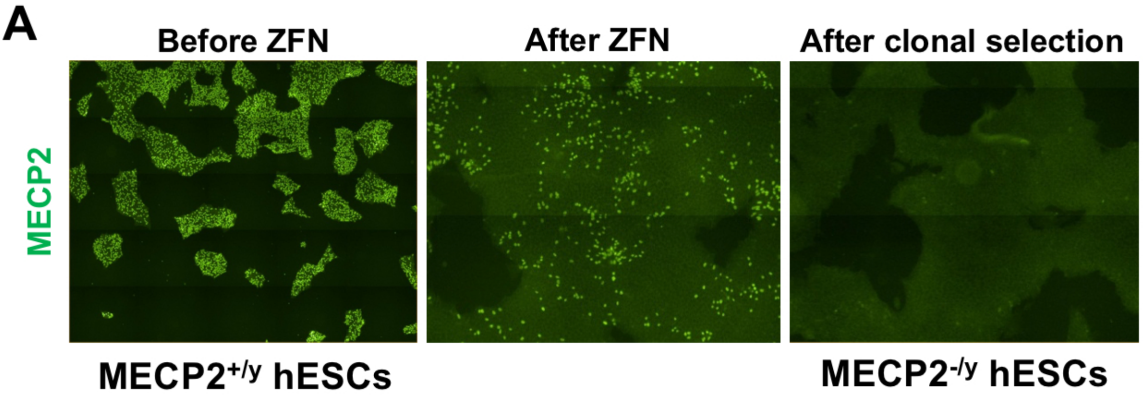

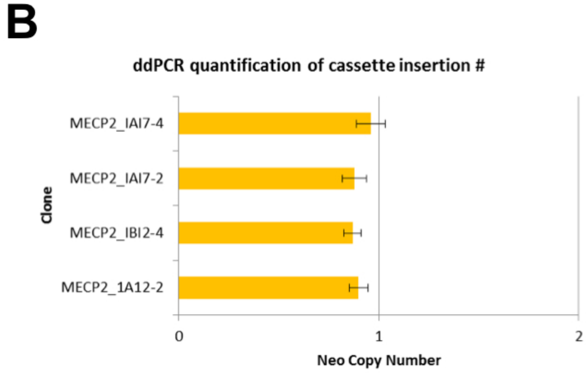

C

Clone	Assay	Copy Number	Poisson error (relative)	Neo copies per haploid genome	Error
MECP2 1A12-2	Neo-FAM	241	0.037	0.9	0.048
MECP2 1A12-2	RPPH1-VIC	538	0.030		
MECP2 IBI2-4	Neo-FAM	320	0.034	0.87	0.043
MECP2 IBI2-4	RPPH1-VIC	737	0.026		
MECP2_IAI7-2	Neo-FAM	159	0.050	0.88	0.060
MECP2_IAI7-2	RPPH1-VIC	361	0.033		
MECP2_IAI7-4	Neo-FAM	96.2	0.060	0.96	0.072
MECP2_IAI7-4	RPPH1-VIC	201	0.040		

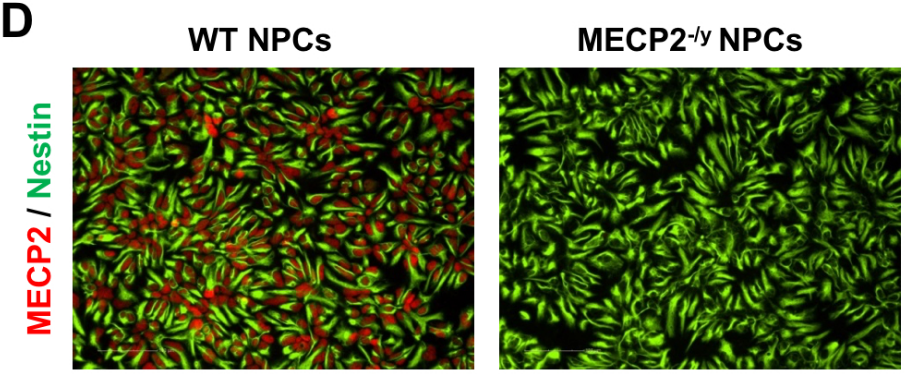

Supplement: S6 Fig — A. Immunofluorescence staining for MECP2 (in green) before ZFN-mediated KO (left panel), after ZFN delivery (middle panel), and after clonal selection (right panel). B. ddPCR graph showing single Neo cassette integration in MECP2-/y hESC clones. C. ddPCR data quantification showing Neo cassette copy numbers per haploid genome and associated data. D. Immunofluorescence staining for MECP2 (red) and Nestin (green) in WT vs MECP2-/y hESC-derived NPCs. (PDF) [file pone.0212553.s006.pdf]
